# Neurofilament Light Chain as a Biomarker in Multiple Sclerosis

**DOI:** 10.3389/fneur.2019.00338

**Published:** 2019-04-05

**Authors:** Kristin N. Varhaug, Øivind Torkildsen, Kjell-Morten Myhr, Christian A. Vedeler

**Affiliations:** ^1^Department of Neurology, Haukeland University Hospital, Bergen, Norway; ^2^Department of Clinical Medicine (K1), University of Bergen, Bergen, Norway; ^3^Neuro-SysMed - Centre of Excellence for Experimental Therapy in Neurology, Department of Neurology, Haukeland University Hospital, and Department of Clinical Medicine, University of Bergen, Bergen, Norway

**Keywords:** neurofilament light (NF-L), biomarker, multiple scleorsis (MS), serum, cerebrospinal Fluid—CSF, axonal damage

## Abstract

Due to the unpredictable course and heterogenous treatment response in multiple sclerosis (MS), there is a clear need for biomarkers that reflect disease activity in the clinical follow-up of these patients. Neurofilaments are neuron-specific components of the cytoskeleton that can be assayed in different body compartments. They have been explored as potential biomarkers for many years. Neurofilament light chain (NF-L) appears the most promising biomarker in MS patients, and there is now little doubt that NF-L should have a role in the follow-up of MS patients. Newer assays and techniques for NF-L detection available in serum samples confirms the usefulness of NF-L as a biomarker. Nevertheless, there is still a need for prospective studies, and studies to determine clinical useful cut-off values. This review evaluates the strengths and weaknesses of NF-L as a biomarker in patients with MS.

## Neurofilaments—an Introduction

Neurofilaments are cytoskeletal components of neurons that are particularly abundant in axons. Their functions include provision of structural support and maintaining size, shape, and caliber of the axons (1). Neurofilaments belong to the intermediate filaments family, and the triplet comprises three subunits; neurofilament light chain (NF-L), neurofilament medium (NF-M) and neurofilament heavy (NF-H). The nomenclature-light (~68 kDa), -medium (~145 kDa),- and heavy (~200 kDa) refers to the molecular weight of the filaments (2, 3).

Following axonal damage in the central nervous system (CNS), neurofilament proteins released into cerebrospinal fluid (CSF) provide an indication of axonal damage and neuronal death. The most extensively subtype studied in this context is NF-L.

Given that neurofilaments are found in the cytoplasm of neurons, all diseases that lead to neuronal and axonal damage can increase the CSF-levels of these proteins. In animal studies, NF-L levels have been used as a marker of axonal damage for decades (4). In humans, neurofilaments were first used as markers of neuronal damage in a study of 12 patients with amyotrophic lateral sclerosis (ALS) and 11 patients with Alzheimer's disease (5).

Subsequently, higher than control levels of neurofilaments were found in the CSF of 60 patients with relapsing-remitting multiple sclerosis (RRMS) (1), suggesting these proteins could also be used as a biomarker of MS disease activity. The field of neurofilament research is rapidly expanding and neurofilament levels are under investigation as markers of disease activity and progression in a number of different neurological conditions including stroke (6), ALS (7), frontotemporal dementia (8), and MS (9). Recently, it has also been suggested that NF-L can be associated with paraneoplastic CNS disorders (10), and peripheral nervous system disorders (11).

Biomarkers of disease activity or disability progression are an unmet need in several neurological conditions. A good and reliable biomarker should say something about the stage of the disease, the prognosis and the response to treatment. A biomarker does not, however, need to be disease specific. It is now clear that elevated NF-L occurs in several neurological disorders and levels depend on age (9, 12). Nevertheless, increased NF-L level appears to reflect ongoing neuronal damage, irrespective of the underlying pathology, making it a potentially interesting biomarker (9).

This review will focus on strengths and weakness of NF-L as a biomarker in the clinical management of MS.

## Multiple Sclerosis—Neurofilaments in Cerebrospinal Fluid and Serum

MS is an inflammatory demyelinating disease of the CNS, usually characterized by relapsing episodes of neurological dysfunction and gradual, progressive decline. Biomarkers reflecting ongoing neuronal damage are therefore of great value in order to characterize the stage of disease, the prognosis and treatment response.

The presence of neurofilament proteins in CSF has been the subject of intense study since the finding of elevated NF-L levels in patients with RRMS (1).

Neurofilament heavy chain has also been investigated as a potential biomarker in MS and levels appear to correlate with Expanded Disability Status Scale (EDSS) in patients with RRMS and clinically isolated syndrome (CIS) (*n* = 102), and also increased in patients with ongoing relapse (*n* = 61) (13). However, comparison of NF-H and NF-L, suggests that NF-L discriminates better between MS (and CIS) and controls (14).

Until recently, the greater majority of studies have focused on detecting these proteins in CSF. Since CSF sampling requires a semi-invasive lumbar puncture, a search for other ways of accessing this clinically useful biomarker has focused on other body fluids. Thus, while lumbar puncture provides a direct approach to the CNS, access to blood is less invasive and better tolerated by patients. Further, as neurofilaments are neuron specific, the finding of these proteins in serum reflects leakage or diffusion through the blood-brain barrier (BBB). NF-L can also reach the blood through CSF drainage into venous blood (15).

Following neuronal biomarkers in blood faces several problems, including patient specific differences in the degree of protein leakage through the BBB (12) and the need for sufficiently sensitive methods of detection. The single-molecule enzyme-linked immunosorbent assay (ELISA) called single molecule array (Simoa) (https://www.quanterix.com) can detect very low concentrations of single enzyme-labeled proteins (16). In fact, this powerful new technique is 126- and 25-fold more sensitive than regular ELISA or electrochemiluminescence, respectively (15).

A strong correlation was detected in paired serum and CSF samples from 373 participants, (286 with MS, 45 with other neurological conditions, and 42 healthy controls) (*r* = 0.62) (17). The important fact that serum NF-L levels correlate with CSF levels have been confirmed in several studies (9, 18, 19). Levels in serum and plasma have also been found to correlate (12). However, although strong positive association is found between CSF and serum NF-L levels, the levels in serum have been evaluated to be a 42-fold lower than in CSF (9).

## Neurofilaments as a Prognostic Biomarker in ON, CIS, and RIS

Patients with either optic neuritis (ON), CIS or radiological isolated syndrome (RIS) are all at risk of developing MS. Identifying those who will convert would be of great importance as one could more aggressively start early treatment. Studies looking at NF-L as a predictor of conversion have provided inconsistent results. In one study investigating multiple biomarkers in ON patients (*n* = 56), increased NF-L levels were found with increasing time from onset to CSF sampling; however, there was no correlation between NF-L and severity of symptoms or Gd-enhanced lesions on MRI (20). Increasing levels of NF-L is interesting in this context as this may reflect the presence of a “silent” axonal damage, and thus neurodegeneration. Subsequently, studies have shown that NF-L could potentially predict conversion to MS after ON (*n* = 86) (21). In addition, CSF NF-L predicted not only visual outcome after ON, but also seemed to have potential as a biomarker for incomplete remission (*n* = 47) (22).

In a retrospective cohort of 68 patients diagnosed with CIS, the levels of CSF-NF-L were significantly higher in patients who later developed MS. However, NF-L was found only to be a weak risk factor for converting to MS compared to oligoclonal bands, and T2 lesions on MRI (23). Another study with a small sample size (CIS patients = 38) sought to evaluate both NF-L and N-acetylaspartate as potential biomarkers. The investigators found higher CSF NF-L levels in both CIS-patients and those in the early stages of MS compared to healthy controls. NF-L levels were also related to conversion from CIS to RRMS (24). This was also the case in a cohort of 109 CIS-patients where converters had higher NF-L CSF levels (25), and in a study of patients with RIS where CSF-NF-L levels were an independent risk factor for later conversion to CIS (*n* = 75) (26). A smaller 2-year follow-up study of CIS patients (*n* = 19), showed higher CSF-NF-L in the converters than non-converters, and NF-L levels further classified 84% of the patients correctly in terms of conversion/non-conversion (27).

In contrast to the above studies, an investigation of 47 patients with CIS confirmed higher levels of CSF NF-L compared to controls, found no difference in NF-L levels between converters and non-converters (28). Likewise, equal CSF NF-L levels were detected in 39 CIS patients, independent of converters or non-converters (29). Yet another study found higher levels of serum-NF-L in CIS-patients compared to controls (*n* = 92), but no difference between what was defined as fast converters to clinical definite MS (*n* = 100) and non-converters (*n* = 98) (30).

Overall, NF-L appears useful as a biomarker and as a predictor of outcome in the early clinical stages prior to definite MS. Although the findings are not completely consistent across studies, this does not invalidate NF-L as a biomarker as the differences could be explained by alterations in diagnostic criteria for CIS, and study design of retrospective studies of relatively small study populations.

## Neurofilaments and Clinical, Radiological, and Prognostic Features in MS

Studies comparing MS patients to healthy controls show that there is a general increase in NF-L levels in patients, and a positive correlation with relapses. Levels of CSF NF-L were almost 10 times higher in MS patients with exacerbations (*n* = 66) than healthy controls (*n* = 50) (31), underlining the correlation between axonal damage and relapses.

When looking at disease progression, correlation of NF-L with EDSS is not always found (31–33). This can, however, usually be explained by small changes in scores over time, small patient populations, limited sensitivity and intra- and inter-rater challenges of the scale. A small study indicated that baseline CSF NF-L levels were higher in those patients that experienced EDSS progression after 5 and 10 years, and was significantly associated with conversion to SPMS (34). The NEDA (no evidence of disease activity)-classification, which comprises EDSS, relapses and MRI changes has also been correlated in various degrees with NF-L (27, 35).

In RRMS and progressive MS patients, the levels of NF-L are higher in the presence of disease activity (*n* = 82) (17). Higher CSF NF-L levels compared to controls were found in primary progressive MS (PPMS), but there was no significant difference between PPMS (*n* = 21) and secondary progressive MS (SPMS) (*n* = 10) (36). In cohort of 99 patients with RRMS, high levels of CSF NF-L were associated with worse outcome and conversion to SPMS (37).

The most objective findings in the clinical follow-up of MS patients are changes in MRI, often evaluated on the basis of new or enlarged T2-lesions, or T1gadolinium-enhanced lesions (Gd-lesions). The studies of radiological changes have also shown more consistent correlations with NF-L levels, and several studies have reported a predictive value in NF-L in regards to one-going lesions, and prior to lesions (9, 17, 27, 32, 38, 39).

A study of two different cohorts, one cross-sectional (*n* = 142) and one longitudinal (*n* = 246) showed generally higher NF-L levels in patients vs. healthy controls, a correlation with presence of relapses, worsening EDSS and MRI lesion activity [both T2-lesions and Gd-lesions (9)]. In another follow-up study of 39 patients, CSF NF-L correctly predicted NEDA-status after 2 years in 85% of cases (27). Both serum and CSF NF-L levels have been found higher during relapses, and with Gd-lesions (17). Further confirmation of these findings came from studies showing positive correlation between serum NF-L and number of Gd-enhanced lesions (*n* = 25) (38), and correlation with new or enlarged T2-lesions and the ability to predict new Gd-lesions (*n* = 85) (32).

In a cohort of 25 natalizumab-treated patients followed for 3 years, CSF-NF-L at baseline correlated with percentage brain volume change (39), and in a retrospective CIS cohort (*n* = 41) NF-L correlated with changes in gray matter on MRI, and an inverse correlation was detected between degree of MRI normalization and NF-L (40).

The radiological correlations with NF-L indicates the usefulness of NF-L as a marker of ongoing brain damage in MS. NF-L is probably the most promising new biomarker to be used in clinical practice for evaluating disease activity.

## Neurofilaments and Treatment Response

Independent of the type of disease-modifying treatment, the majority of investigators have detected an inverse correlation between NF-L levels and treatment. Lower levels of NF-L were found in treated patients compared to treatment-naïve individuals (*n* = 21) (38), and it has also been shown that NF-L levels fall in follow-up studies of disease-modifying treatment vs. no treatment (9). CSF and serum-NF-L levels are stable in treatment-naïve patients (*n* = 10) or when shifted to similar efficacy (*n* = 20), but fall when patients are shifted to drugs with higher efficacy (*n* = 68) or when drugs are started in the treatment-naïve (*n* = 50) (17). Serum NF-L levels fall after initiation of interferon-beta (*n* = 85) (32), and plasma NF-L levels fell by 34% after 12 months of fingolimod treatment (shifted from interferon or glatiramer acetate), (*n* = 243) (12).

In a cohort of 92 MS patients started on natalizumab, CSF collected at baseline and after 6 or 12 months showed a significant fall in NF-L, to similar levels as healthy controls, independent of relapses in the months before treatment startup (41). These findings have been confirmed in other studies (33), and also in fingolimod-treated patients vs. placebo in CSF (*n* = 36) (42). In one cohort of 75 patients with clinical stable RRMS, patients were switched from first-line injectable treatment to rituximab and followed with CSF-NFL at baseline, at month 12 and month 24 (*n* = 65). NF-L levels decreased significantly 12 months after therapy shift, and as clinical and radiological signs from relapse approached at 24 months, NF-L also increased, but not significantly, indicating NF-L as a marker of treatment response (43).

In a study of 59 MS patients on either interferon-beta (*n* = 33) or natalizumab (*n* = 19), or without treatment (*n* = 7), CSF NF-L levels were lower in both treatment groups, but the natalizumab-group was not significantly different from healthy controls, and the interferon-beta NF-L levels were still significantly higher than in the natalizumab-group (44).

In 35 patients with progressive MS, CSF NF-L levels were evaluated after 12–24 months of mitoxantrone or rituximab treatment and showed a significant decrease. For the patients on disease modifying agents at baseline, the NF-L values were already lower than the untreated group (45).

There is now clear evidence that NF-L is a good biomarker for treatment response in MS, especially for high efficacy drugs. This is probably due to the better prevention of brain damage in these treatments, underlining the role of NF-L as a marker of neuronal and axonal damage.

## Neurofilaments in General—What Next and How to Use NF-L in Clinical Practice?

The majority of studies that have specifically looked at NF-L have not been performed prospectively, but retrospectively on samples taken at varying time points in relation to clinical symptoms. Further, study populations have often been small. Recently, a meta-analysis of results from 15 studies verified a significant increase of NF-L in MS patients compared to controls (46). Still, prospective studies of much larger cohorts focusing on NF-L and collecting baseline and follow-up data are necessary.

Before taking the Simoa-assay in to clinical use it is necessary to compare the results of the assay between different centers (15). Determining age-dependent cut-off values are also necessary.

A recent study aimed to estimate percentile curves for healthy controls across different age group to be used as reference (9). In serum samples from 246 healthy controls (median age 44.3 years), the median serum NF-L was 22.9 pg/ml (9). Of these, 87 controls had 1-year follow-up serum sample, and the median NF-L level increased with 1.8%, fitting with the observed positive age-association seen in the whole cohort, where the increase was 2.2% for each additional year (9). The median NF-L value found in the healthy controls is in accordance with healthy controls in a heterogenous group of studies summarized in Table 1. Taken together, it seems that a serum NF-L value of 16–20 pg/ml is normal in healthy individuals, with age being an increasing factor.

**Table 1:** Serum NF-L levels in a heterogenous group of healthy controls.

**Examined disorder**	**HC (*n*)**	**HC age**	**HC serum NF-L (pg/ml)**	**Patient serum NF-L (pg/ml)**	**Assay**	**References**
ALS	50	55	16.2	125	Simoa	(47)
ALS	12	47	17	255/196 (early/late symptomatic phase)	Simoa	(7)
ALS	19	33	7.5	>54.5	ECL	(48)
AD	12	86	29	42	Simoa	(49)
AD dementia	193	76	34.7	AD dementia 51.0	Simoa (plasma)	(18)
				MCI 42.8		
FTD	28	65	19.6	77.9	Simoa	(50)
FTD	73	NA	3.5	31.5	ECL	(8)
CIS	92	35	7.9	24.1/19.3(Converter/NC)	ECL	(30)
MS	22	32	11	17	Simoa	(35)
MS	42	28	10.5	16.9/23 (RRMS/PMS)	Simoa	(17)
PN	25	NA	6.91	31.5	Simoa	(11)
PSP	12	70	17.5	31	Simoa	(51)
Traumatic brain injury	35	31	13	>90	Simoa	(52)
Concussion	142	NA	8.47	NA	Simoa	(53)

*HC, healthy controls; ALS, amyotrophic lateral sclerosis; AD, Alzheimer's disease; MCI, mild cognitive impairment; FTD, frontotemporal dementia; ECL, electrochemiluminescence; CIS, clinically isolated syndrome; NC, non-converters; PN, peripheral neuropathies; PSP, progressive supranuclear palsy*.

Interestingly, MS is not the neurological disorder that gives the highest values of NF-L (Table 1). Both ALS (7, 47, 48) and Creutzfeldt-Jacobs disease (54) are examples of disorders that gives especially high NF-L levels. These disorders are characterized by neurodegeneration, and little inflammation, which suggests that increased NF-L levels in active MS patients, not only reflects inflammation, but also an ongoing neurodegeneration.

NF-L cannot be used as a specific diagnostic tool in MS but, combining NF-L with other biomarkers is of value. In one cross-sectional study of 271 patients with clinical features of suspected MS onset, the combination of CSF NF-L and intrathecal immunoglobin G production had a sensitivity of 97% for detecting RRMS patients (55).

Despite not being a MS-specific biomarker, NF-L does appear to be a useful marker for disease activity and treatment response. There is good evidence that NF-L has a role in everyday clinical follow-up of MS patients, particularly as a marker of subclinical activity in RRMS (17, 32).

The question now is how to best use NF-L in clinical practice. A recent study showed that the early serum NF-L levels in newly diagnosed MS can potentially predict lesion load and brain atrophy on MRI after 10 years (56). This leads us to speculate if one should be more aggressive in treating patients with high serum NF-L levels at time of diagnosis, which, brings us to the need of cut-off values to determine high, medium and low NF-L levels.

In the follow-up of MS patients, NF-L cut-off values may be important, i.e., does the level in healthy people have a significance, or should we solely use the intra-individual value? We would suggest the latter. MS patients should be monitored with serum levels of NF-L with a time-interval of perhaps 3–6 months depending of the course of the disease. If the serum level of NF-L increases, the clinical activity should also be evaluated by MRI. In this way, unnecessary routine MRI screening can be avoided and disease activity more reliably detected. In a 2-year follow-up study of RRMS patients, an individual increase of 10 pg/ml in serum NF-L levels gave increased risk of new T1 gadolinium-enhanced lesions, and new T2 lesions (32). This increase was higher than the expected age-related increase in yearly follow-up, and might therefore indicate a level to use as an individual cut-off. What is less clear however, is whether a similar decrease in NF-L levels of 10 pg/ml is of any clinical interest. Using NF-L rate of change as a marker for disease progression is in terms with a recent study in dominant inherited Alzheimer's disease, where the rate of change in NF-L levels was associated with degree of cortical thinning on MRI (19).

In conclusion it is our opinion that serum NF-L measurements should systematically be used as prognostic biomarker to monitor MS patients for progression, disease activity, and treatment efficacy.

## Author Contributions

All authors listed have made a substantial, direct and intellectual contribution to the work, and approved it for publication.

### Conflict of Interest Statement

The authors declare that the research was conducted in the absence of any commercial or financial relationships that could be construed as a potential conflict of interest.
